# Possible Brucellosis in an Early Hominin Skeleton from Sterkfontein, South Africa

**DOI:** 10.1371/journal.pone.0006439

**Published:** 2009-07-30

**Authors:** Ruggero D'Anastasio, Bernhard Zipfel, Jacopo Moggi-Cecchi, Roscoe Stanyon, Luigi Capasso

**Affiliations:** 1 State University “G. d'Annunzio”, Department of Human Movement Sciences, Section of Anthropology, Chieti, Italy; 2 University of the Witwatersrand, Bernard Price Institute for Palaeontological Research and Institute for Human Evolution, Johannesburg, South Africa; 3 University of Florence, Department of Evolutionary Biology, Laboratories of Anthropology, Florence, Italy; Indiana University, United States of America

## Abstract

We report on the paleopathological analysis of the partial skeleton of the late Pliocene hominin species *Australopithecus africanus* Stw 431 from Sterkfontein, South Africa. A previous study noted the presence of lesions on vertebral bodies diagnosed as spondylosis deformans due to trauma. Instead, we suggest that these lesions are pathological changes due to the initial phases of an infectious disease, brucellosis. The macroscopic, microscopic and radiological appearance of the lytic lesions of the lumbar vertebrae is consistent with brucellosis. The hypothesis of brucellosis (most often associated with the consumption of animal proteins) in a 2.4 to 2.8 million year old hominid has a host of important implications for human evolution. The consumption of meat has been regarded an important factor in supporting, directing or altering human evolution. Perhaps the earliest (up to 2.5 million years ago) paleontological evidence for meat eating consists of cut marks on animal remains and stone tools that could have made these marks. Now with the hypothesis of brucellosis in *A. africanus*, we may have evidence of occasional meat eating directly linked to a fossil hominin.

## Introduction

The study of pathology in ancient human and pre-human populations can contribute to our knowledge of their life history and health status. Paleopathological investigations have the potential to yield particularly noteworthy results when the subjects of study are hominins. The documented array of pathological lesions affecting *Australopithecus* and early *Homo* consists of trauma, malformations, tumours, degenerative alterations associated with ageing and bipedal locomotion [1]–[6]. To date, there are no descriptions of infectious disease in early hominins and we could only speculate on what infectious organisms may have affected australopiths. Here we present the first possible case of an infectious pathology in an australopith (Stw 431) from the hominin site of Sterkfontein South Africa. Stw 431 was recovered *in situ* from Member 4, Bed B, of the Sterkfontein Formation [7]. Member 4 was previously estimated to be 2.4–2.8 Ma [8]–[11], while Berger et al. [12] later placed it at between 1.5–2.5 Ma. On grounds of provenance and compatible morphology, Stw 431 is attributed to *Australopithecus africanus* [7]. The skeleton is that of an adult individual, probably male, and is comprised of eighteen mostly incomplete bones derived from the axial skeleton, pectoral girdle, upper limb, and the pelvic girdle. The vertebral column consists of nine consecutive thoracolumbar vertebrae, T9 to L5.

Lumbar vertebra L5 presents lytic lesions that Staps [13] described as spondylosis deformans resulting from a trauma. Here we discuss the possibility that these lytic lesions may be more consistent with pathological skeletal changes possibly due to brucellosis. Our findings support the hypothesis of a more complex diet of *A. africanus* that occasionally could have included meat to supplement its diet [14].

### Analysis

The fossil lumbar vertebrae L4 and L5, were examined with a stereomicroscope (Leica Wild M8) at the School of Anatomical Sciences at the University of the Witwatersrand. A mould of the lytic lesions of the anterior superior margin of the vertebral bodies of L4 and L5 was made using “President Plus Jet”, type 3, and “Elite H-D +”, Types 2 and 3. Casts were then produced and observed with a Scanning Electron Microscope (SEM) to examine the presence of osteoclastic and osteoblastic activity, which would suggest that inflammatory processes were in progress at the time of death of the individual. The SEM analyses were conducted in the Laboratories of the State University “G. d'Annunzio” in Chieti, Italy. Plain film radiographs were taken at the Helen Joseph Hospital in Johannesburg, South Africa.

## Results

A preliminary examination revealed the presence of some pathological lesions on the vertebral bodies. Lumbar vertebrae L4 and L5 have lytic lesions on the superior-anterior margin of the vertebral bodies; in particular L5 showed an excavation of the anterior-superior body with osteophytes. The position and gross morphology of the lesions were very similar to the pathological bone alterations observed in some infectious diseases in modern humans, such as in human zoonotic brucellosis [15].

**Figure 1:**
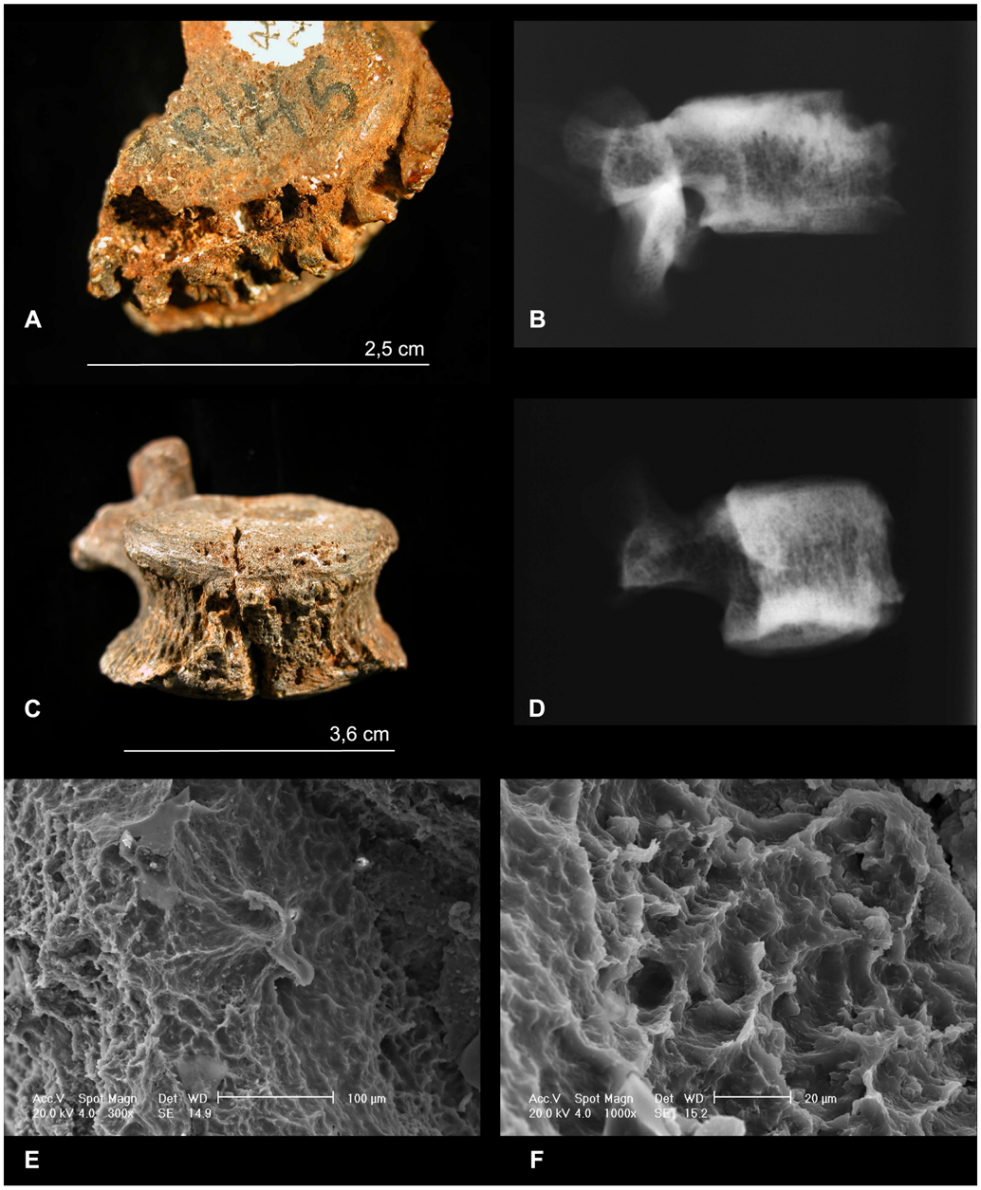
Lumbar vertebrae L5 and L4 of *Australopithecus africanus* Stw 431. Stereomicroscopic and radiographic studies of L5 (A, B), and L4 (C, D). Scanning Electron Microscope (SEM) analysis showing Howship's lacunae of L5 (E) and L4 (F).

The lumbar vertebra L5 of Stw 431 has a destructive focus on the superior-anterior margin of the body, with clear signs of active bone reaction (Figure 1A). Scanning Electron Microscope (SEM) analysis of the trabeculae surrounding the walls of the lytic lesion revealed sheets of new bone formation and Howship's lacunae due to the osteoclastic and osteoblastic cell activity (demonstrating that the bone alteration occurred *in vitam*) (Figure 1E). The lateral radiograph shows the destructive lesion of the anterior vertebral body and a sclerosis limited to the affected area consistent with the *Sign of Pedro-Pons* [16], [17] (Figure 1B).

The lumbar vertebra L4 of Stw 431 presented granulomatosic tissue in a clearly delimited region of the anterior rim of the body (Figure 1C). The SEM images revealed bone destruction mediated by osteoclasts (Figure 1F), whose activity may have been stimulated by pathogenic organisms. The radiographs of the sample also revealed a sclerotic repair of the lesion (Figure 1D).

## Discussion

The macroscopic, microscopic and radiological appearance of the lytic lesions of 4th and 5th lumbar vertebrae is consistent with all the skeletal characteristics of brucellosis. The remaining vertebrae and bones of this australopith skeleton did not show any other pathological lesions or degenerative changes.

**Figure 2:**
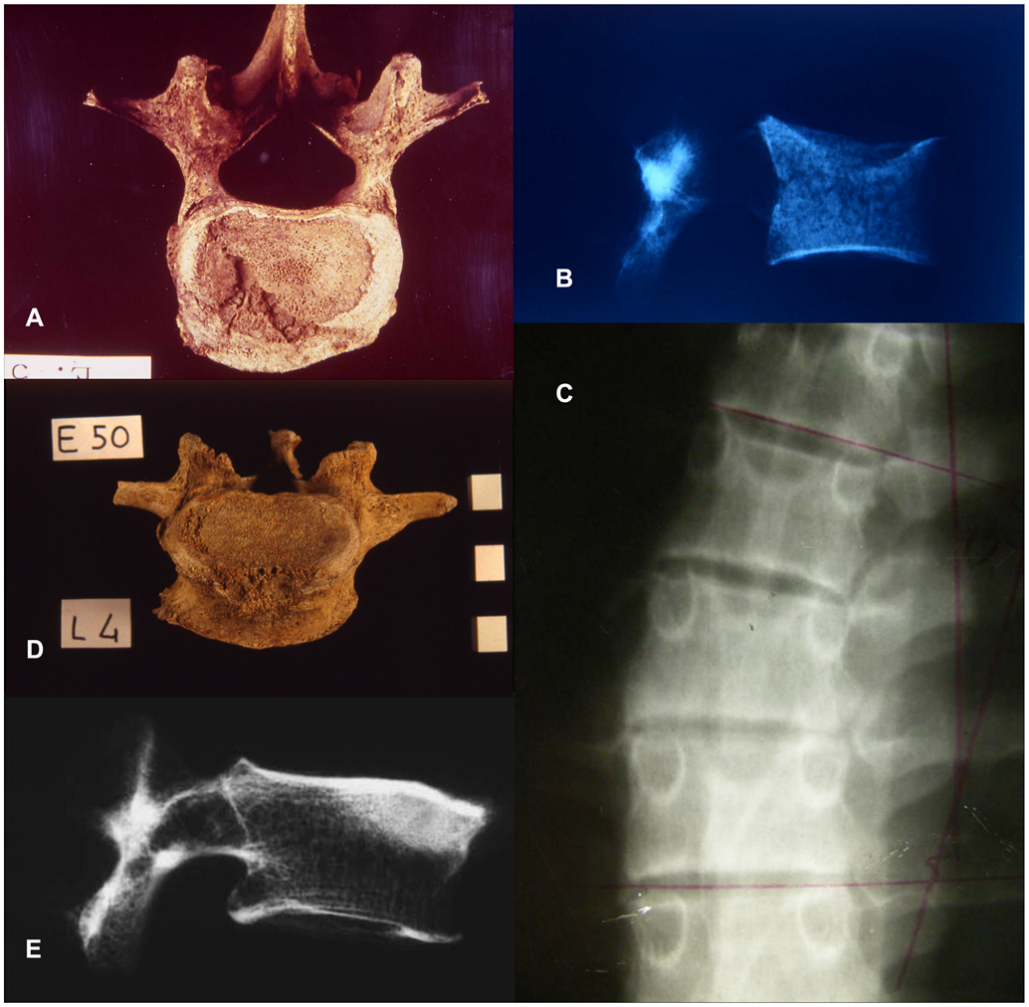
The lytic lesions on Stw's 431 L4 and L5 were compared with ancient modern human lumbar vertebrae affected by brucellosis and tuberculosis, and with Scheuermann's disease in a living young male. Modern human L4 affected by tuberculosis: macroscopic (A) and radiographic picture (B) [18] (Courtesy of Capasso; adult female E58, Herculaneum, 79 AD, University Museum of Chieti). Scheuermann's disease in the vertebral column of a living 18 years old male: Radiographic analysis (C) (Courtesy of Zipfel; School of Anatomical Sciences at the University of the Witwatersrand). Modern human L4 affected by brucellosis: macroscopic (D) and radiographic picture (E) [18] (Courtesy of Capasso; adult female E50, Herculaneum, 79 AD, University Museum of Chieti).

We carefully considered alternative diagnoses of various infectious diseases including tuberculous and staphylococcic spondylitis, which in modern humans occasionally develop anterior epiphysitis of the vertebral body [18]. Nevertheless these diseases seemed less likely to produce the complex of alterations we observed. For instance, unlike early brucellosis that frequently affects the lumbar column and particularly L4 and L5, these disease agents are usually not circumscribed and frequently involve other vertebrae [16]. In general, tuberculosis of the spine results in collapse of the vertebral bodies and angular deformity [19]–[22] (Figure 2A, B ), conditions that are absent in the spine of Stw 431. We also considered Scheuermann's disease which in 1983 Cook et al. [3] diagnosed in the vertebral column, more specifically T8 of the australopith AL-288-1AG. However, this juvenile pathology, typically affecting the thoracic vertebrae, shows lesions that differ from brucellosis both morphologically and radiologically (Figure 2C).

We also evaluated the possibility that the marked epiphysitis observed in the L5 of Stw 431 may have resulted from degenerative processes due to aging and/or bipedal locomotion reported in early hominins. However, this possibility also seemed less likely because the paleopathological examination of the skeletal elements of Stw 431 did not show any other degenerative lesions that could be associated with age or mode of locomotion.

Staps [13] described the vertebral lesions in the Stw 431 individual as spondylosis deformans of L5 as a result of trauma. The lytic lesion of the superior-anterior margin of L5 may be interpreted as a lesion having resulted from an intrabody herniation of disc material (so-called Schmorl's node) [23] or as the sequela of a remote injury in an immature skeleton (limbus vertebrae) [24]–[26]. Trauma, directly or with possible consequent necrosis of the anterior epiphysis is a possible interpretation that cannot be completely ruled out. However trauma does not appear to fit well with the lesion in L4: the granulomatosic tissue in the delimited region of the anterior rim of the body was more likely the result of an acute inflammatory process rather than of a traumatic fracture of the superior-anterior margin of the vertebral body.

After carefully evaluating all reasonable alterative hypotheses we suggest that the position, gross morphology and the radiological appearance of the lesions observed in the vertebral elements L4 and L5 of *Australopithecus africanus* Stw 431seem to be more consistent with the pathological condition of early brucellosis [15].

Brucellosis in humans is currently comparatively rare in developed countries [27]. Many recent cases come from areas such as North Africa and the Middle East. The disease in humans occurs as a chronic infection of the lungs and other organs. In a number of cases, the skeleton becomes infected by the hematogenous route [28]. Skeletal involvement varies from 2% to 70% of the cases [29] and 20% to 80% of patients with brucellosis experience osteoarticular symptoms [30]. In historical times adult human males are affected much more frequently than females, independently from the species of *Brucella* involved [31]. The most common skeletal lesion occurs in the spine or the sacroiliac joint [22], [32]. Ganado and Craig [33] observed 130 instances of spondylitis in 6300 patients with brucellosis. Long bones were rarely involved [19]. Kelly et al. [34] observed in 36 cases the following distribution: spine, 47,2%; humerus, 8,3%; femur, 5,6%; ilium, 2,8%; hand, 2,8% and foot, 2,8%. The spinal lesions involved the vertebral bodies, especially of the lower thoracic, lumbar and lumbosacral areas [22], [35], [36]. The early skeletal signs of brucellar diseases are characterised by the epiphysitis of the anterior-superior angle of the lumbar vertebrae, radiographically showing a selective sclerosis in the antero-superior angle of the vertebra. This radiographic expression is known as the *Sign of Pedro-Pons* and represents a characteristic aspect of the early stage of brucellar disease in ancient human remains [16], [17], [37]–[43]. Brucellar lesions on vertebral bodies were observed also in human skeletal remains from archaeological sites [15], [18], [44]–[47] (Figure 2D, E).

In humans, *Brucella melitensis* is the main etiological agent of brucellosis and its diffusion is linked to the consumption of milk and dairy products such as unpasteurised cheese; yet other bacterial species such as *Brucella abortus*, may be transmitted to humans and other primates [48]. *B. abortus* usually leads to abortion in the host and the infection is transmitted through contact with foetal membranes, post parturient discharges and milk [49]. In recent times *B. abortus* has been isolated from several South African species of wildlife such as zebra (*Equus burchellii*), eland (*Taurotragus oryx*), waterbuck (*Kobus ellipsiprymnus*) and impala (*Aepyceros melampus*) [49].

Given this background, it seems reasonable to suggest that some species of *Brucella* (possibly even *B. abortus*) could have been the infective agent in this *Australopithecus africanus* individual, through contact with (or consumption of ) infected tissues of other mammals, such as parturient discharges, foetal membranes or meat of young antelopes or other Ungulata. Occasional meat eating is well documented among baboons and chimpanzees [50]–[53] and therefore similar dietary behaviour in australopith from South Africa seems likely [54]. Our hypothesis is supported by the results of stable carbon isotope analysis of *A. africanus* from Makapansgat Limeworks, South Africa, that suggest a diet based not only on fruit and leaves, but also on large quantities of carbon-13-enriched foods such as grasses and sedges or animals that ate these plants, or both [55], [56].

In conclusion, we feel that the type and distribution of the lytic lesions observed in the lumbar vertebrae L4 and L5 of the Stw 431 individual are most consistent with early brucellosis. Consequently, this paleopathological case may represent the first example of an infectious disease in an australopith and, in particular, suggests that mammal tissues could have been an important but perhaps highly variable part of the australopith diet. However, even in light of this compelling evidence we strike a cautionary note as we cannot completely rule out that these lesions result from other infectious diseases such as echinococcosis and fungal diseases [19] or even an unusual form of trauma.

The hypothesis of brucellosis (most often associated with the consumption of animal proteins) in a Pliocene hominin has important implications for human evolution. The consumption of meat has been regarded an important factor in supporting, directing or altering human evolution [57]–[61].

Perhaps the earliest paleontological evidence (up to 2.5 m.y. BP) for meat eating consists of cut marks on animal remains and stone tools that could have made these marks [62]–[65]. Now with the hypothesis of brucellosis in *A. africanus* we may have evidence of occasional meat eating directly linked to a fossil hominin.
